# Critical Characteristics of Housing and Housing Supports for Individuals with Concurrent Traumatic Brain Injury and Mental Health and/or Substance Use Challenges: A Qualitative Study

**DOI:** 10.3390/ijerph182212211

**Published:** 2021-11-20

**Authors:** Maria Jennifer Estrella, Bonnie Kirsh, Pia Kontos, Alisa Grigorovich, Angela Colantonio, Vincy Chan, Emily Joan Nalder

**Affiliations:** 1 Department of Occupational Science & Occupational Therapy, University of Toronto, Toronto, ON M5G1V7, Canada; bonnie.kirsh@utoronto.ca (B.K.); angela.colantonio@utoronto.ca (A.C.); emily.nalder@utoronto.ca (E.J.N.); 2 Rehabilitation Sciences Institute, University of Toronto, Toronto, ON M5G 1V7, Canada; vincy.chan@uhn.ca; 3 Department of Psychiatry, University of Toronto, Toronto, ON M5T 1R8, Canada; 4 KITE Research Institute, Toronto Rehabilitation Institute—University Health Network, Toronto, ON M5G 2A2, Canada; pia.kontos@uhn.ca (P.K.); agrigorovich@brocku.ca (A.G.); 5 Dalla Lana School of Public Health, University of Toronto, Toronto, ON M5T 3M7, Canada; 6 Department of Recreation and Leisure Studies, Brock University, St. Catharines, ON L2S 3A1, Canada; 7 Institute of Health Policy, Management and Evaluation, University of Toronto, Toronto, ON M5T 3M6, Canada

**Keywords:** housing, homelessness, traumatic brain injury, mental illness, substance use, concurrent disorders, health services, qualitative

## Abstract

Traumatic brain injury (TBI) and mental health and/or substance use challenges (MHSU) are commonly co-occurring and prevalent in individuals experiencing homelessness; however, evidence suggests that systems of care are siloed and organized around clinical diagnoses. Research is needed to understand how housing and housing supports are provided to this complex and understudied group in the context of siloed service systems. This study aimed to describe critical characteristics of housing and housing supports for individuals with concurrent TBI and MHSU from the perspectives of service users with TBI and MHSU and housing service providers. Using basic qualitative description, in-depth interviews were conducted with 16 service users and 15 service providers. Data were analyzed using thematic analysis techniques. Themes capture core processes in finding and maintaining housing and the critical housing supports that enabled them: (1) overcoming structural barriers through service coordination, education and awareness raising, and partnerships and collaborations; and (2) enabling engagement in meaningful activity and social connection through creating opportunities, training and skills development, and design of home and neighborhood environments. Implications for practice, including the urgent need for formalized TBI and MHSU education, support for service providers, and potential interventions to further enable core housing processes are discussed.

## 1. Introduction

An estimated 150 million people worldwide are experiencing homelessness, a global problem considered to be the most extreme manifestation of housing and social exclusion [1,2]. Canada is no exception, with more than 235,000 individuals experiencing homelessness in any given year and 35,000 on any given night [3]. In this paper, we use the term homelessness to refer to a range of living situations, including unsheltered or living on the streets, emergency sheltered, and temporary accommodation and to consider those individuals currently experiencing, or at risk of, homelessness [4]. Among individuals experiencing homelessness, there is a high prevalence of traumatic brain injury (TBI) and mental health and/or substance use challenges (MHSU) [5,6,7]. TBI and MHSU are often co-occurring conditions that are associated with housing instability and prolonged homelessness [8,9,10]. Addressing homelessness in the long-term necessitates safe, affordable, and appropriate housing and housing support services [11]. As such, understanding critical characteristics of housing (i.e., valued characteristics of the dwelling and the neighborhood), and housing supports (i.e., services or supports for housing and other areas of life) is an important step in enabling individuals with co-occurring TBI and MHSU to find and maintain housing. 

In recent years, Housing First has emerged as the recommended approach to address homelessness in many parts of the world, including Canada. Housing First is considered as an *intervention* designed to meet the needs of individuals/groups experiencing homelessness, and as a *philosophy* that can guide how housing and other services are considered and organized [12]. Philosophically, Housing First is guided by core principles, including immediate provision of housing and consumer-driven services, de-linking of housing and clinical services, providing recovery-oriented supports, and facilitating community reintegration [13]. In essence, it calls for person-centered and holistic services that prioritize consumer choice and facilitate access to housing while also linking individuals with services that will meet other needs including those related to health and community integration. 

While Housing First is associated with positive outcomes, such as housing stability, improved quality of life and community functioning, and reduced service use [14,15], having a history of TBI is one factor associated with lower well-being among those accessing Housing First services [16]. Additionally, individuals with concurrent TBI and MHSU had different patterns of service use to those with MHSU and no TBI, including greater interaction with emergency and family medicine and criminal justice services [7]. This suggests that those with concurrent TBI and MHSU may have different needs in terms of housing and support services that need to be understood. 

Research has shown that individuals with TBI and MHSU report poorer psychological and physical health and greater unmet care needs than individuals without co-occurring conditions [17,18]. Certain barriers to providing optimal services for those with TBI and MHSU who are experiencing homelessness have been proposed, including: (1) limited evidence for interventions (i.e., how to support individuals with concurrent TBI and MHSU with housing); (2) a lack of knowledge of TBI among professionals in housing or mental health services; and (3) having multiple concurrent conditions (i.e., TBI and MHSU) affecting eligibility for services [19,20,21]. These barriers speak to a complex service environment for this group. Further, an environmental scan of Canadian integrated care systems found that individuals with concurrent TBI and MHSU received supports through a brain injury program, and mental health or substance use care was provided by a separate stand-alone program [18]. In this way, individuals must access care across a siloed system organized around diagnoses. As this work was not specific to housing, it will be important to examine how housing supports are provided and experienced by those with TBI and MHSU. Numerous calls for research and specialized services have been made to better serve individuals with TBI and MHSU [7,18,19,20]. However, there continues to be a knowledge gap in what individuals with concurrent TBI and MHSU who are receiving housing services consider critical characteristics of housing and housing supports.

Qualitative research can illuminate the perspectives of those providing or receiving services regarding the critical characteristics of housing and housing supports. Despite the frequent co-occurrence of TBI and MHSU, housing research has studied TBI and MHSU separately, with greater attention to the perspectives of individuals with MHSU. For example, several qualitative studies and reviews in the MHSU literature revealed the following key characteristics of housing and supports: (1) the neighborhood and proximity to services and outdoor environments; (2) having choice regarding housing; (3) an environment that promotes feelings of safety, security, and comfort; and (4) services that attend to other aspects of life including participation in activities, neighbor and social contact, education (e.g., life skills workshops), and healthcare needs (i.e., counseling, substance use and other mental health services, and intensified mental health support during times of crisis) [22,23]. A systematic review identified housing features (e.g., physical access, homely environment, safety, and security) relevant to individuals with neurological disabilities but did not tease out the perspectives of individuals with TBI specifically [24]. A qualitative study reported challenges with finding appropriate living environments for individuals with TBI, however, it did not explore the key characteristics of housing or housing supports, and reported only the perspective of service providers, not individuals with TBI themselves [25]. More research is needed to understand the characteristics of housing and housing supports that enable individuals to find and maintain housing, from the perspective of individuals with concurrent TBI and MHSU and those providing services in this context.

In summary, recommended approaches to address homelessness, such as Housing First, call for the provision of housing and appropriate supports to address individuals’ needs holistically, including housing, health, and community integration. Despite the frequent co-occurrence of TBI and MHSU [7] and the complex siloed systems that characterize the service environment for this group [18,20,21,26], there is limited research discussing the key characteristics of housing and housing supports for those with TBI and MHSU. Research that studies TBI and MHSU in isolation of each other implies that individuals who have concurrent TBI and MHSU require complex care and yet “fall into the cracks,” unable to obtain the appropriate housing and housing supports that would help them secure housing and stay housed. The perspectives of those with concurrent TBI and MHSU and services providers will provide a deeper understanding of how and why services are provided in complex environments, and illuminate the kinds of services that exist and work well, as well as services that require improvement. 

This study aims to explore the critical characteristics of housing and housing supports from the perspective of both individuals with concurrent TBI and MHSU (hereafter referred to as “service users”) and service providers in an urban Canadian location with publicly funded healthcare.

## 2. Materials and Methods

### 2.1. Design

The study was guided by a “basic qualitative description” approach as outlined by Sandelowski [27] and Kim and colleagues [28]. This approach was fitting, as it allowed us to capture in depth participants’ experiences and offered a rich descriptive account of critical characteristics of housing and housing supports [27]. 

### 2.2. Participants and Recruitment

Two groups of participants—service users and service providers—were recruited using purposeful sampling techniques in collaboration with community agencies providing housing services to individuals with TBI and MHSU in the Greater Toronto Area (GTA) [29]. All provided a range of housing services including supportive housing with housing supports, short-term residential services and supports, and case management services. Some community agencies were specialized and provided supports to either adults living with TBI (*n* = 2) or MHSU (*n* = 4) or both (*n* = 1). Service providers from these community agencies facilitated recruitment by: distributing posters in dedicated sites and drop-in centers; circulating flyers that have information about the study to staff and clients via newsletters and email; and explaining the study to eligible individuals accessing their services. Individuals interested in the study then contacted the first author who provided detailed information about the study and confirmed their eligibility to participate. Written informed consent was obtained prior to the interview. This study was approved by the Research Ethics Board of the University of Toronto (protocol #36009). 

To participate, service users had to (1) be accessing or on a waitlist for housing services; (2) have a diagnosed mental illness and/or substance use disorder or be receiving services for documented concerns regarding MHSU; (3) have a diagnosed TBI or self-identify as having a lifetime history of TBI ascertained using questions from the Ohio State University Traumatic Brain Injury Identification Method (OSU TBI-ID) [30]; and (4) be aged 18 years or over and able to communicate in English. Service providers were eligible if they (1) were working in housing services for individuals with TBI, MHSU, or both; (2) had at least 6 months of experience working with individuals with TBI and/or MHSU; and (3) were aged 18 years or over and able to communicate in English. 

### 2.3. Data Collection

Data were collected using a demographic form and in-depth semi-structured interviews. Participants were asked to complete the demographic form before beginning the interview. Service users were asked about demographic information (e.g., age, gender), their social situation (e.g., source of income, living situation), and health status (diagnosis/self-report of TBI and diagnosis of mental illness and/or substance use disorder or documented MHSU), while service providers were asked for demographic characteristics, information about their current job (e.g., job title, length of employment), and their work history/experience. 

In-depth semi-structured interviews were conducted by the first author in either a private room at the University of Toronto, the community agency, or a common area in the participant’s residence. Participants were reminded of the goal of the study to provide context to the interview questions. Open-ended questions that explored participants’ experiences throughout their housing journey were asked first, followed by probing questions regarding experiences of TBI or MHSU as related to housing and housing supports. The primary question was “Can you tell me about your current housing situation and how you got there?” followed by specific questions such as:

- Can you tell me about the challenges you experienced that affected your housing situation along the way?
- What helped you get into and settled into your current housing situation?
- What supports do you have that help you maintain this living situation?
- Is there anything else you wish you had help with?
- What are your hopes for the future regarding housing?
- What suggestions would you like to offer to improve housing for people with brain injury and [mental health and/or substance use] challenges?

Service providers were asked to describe their role and experience providing housing supports for service users and the kinds of supports that help them find and maintain housing. Probing questions included challenges they experience when providing these supports and supports needed to address those challenges. Examples of questions included:

- What supports does your organization provide to help individuals with TBI and [mental health and/or substance use] challenges find and maintain housing?
- What do you think are the major challenges that this group face in finding and maintaining housing?
- What are the challenges you face in providing housing supports for this group?
- What services and supports are in place or are needed to address these challenges?

In total, 31 participants were interviewed, with each interview lasting 1–2 h. We found the final sample to be adequate based on the commonalities, variations, and amount of detail and nuance in participants’ experiences of housing services [31].

### 2.4. Data Analysis

Data were analyzed using thematic analysis, specifically “codebook” thematic analysis [32,33]. This was chosen as the method of analysis for the following reasons: it aligns with basic qualitative description by identifying common threads (i.e., critical characteristics of housing and housing supports) while staying as close as possible to participants’ own words/perspectives [28,34]; it facilitated the identification of similarities and differences in the perspectives of service users and service providers [35]; and it is fitting for applied research with accessible and actionable findings [32].

The analysis process involved a series of activities to identify and report patterns in the data and their meaning in terms of understanding critical characteristics of housing and housing supports. Interviews were transcribed by an external transcription service and checked by the first author. To become familiar with the data, the first author listened to audio recordings of interviews, read transcripts as they were completed, and wrote reflections on ideas generated from engaging with the data. To identify patterns/themes, research team members reviewed and coded transcripts and engaged in biweekly discussions about coding and the first authors’ memos/reflections completed during analysis. In terms of coding, five members of the research team read and coded 3–4 transcripts independently. Observations and ideas were then discussed in multiple team meetings and synthesized by the first author, leading to the development of a coding framework. A second analyst who was not involved with recruitment or data collection was engaged in this stage to test and refine the coding framework. Upon refinement, the first author applied the codes from the framework to all transcripts using NVivo 12(QSR International, Burlington, Massachusetts, USA). In applying the framework, additional modifications were made as needed, including adding new codes, creating higher order codes, and revising the framework as new ideas were generated. Themes were developed by reviewing and discussing the coded transcripts and grouping common ideas together. Other analytic strategies were used to develop themes, including: discussions with the research team asking questions of the data (e.g., How are housing services provided and experienced? What are service providers addressing with their clients and why? How are housing and housing supports thought about?); engaging with the literature; and writing and checking developed themes in terms of how they reflected ideas generated through the initial stages of reading and coding of transcripts, and how they relate to the research aim.

Researchers involved in data analysis had different disciplinary backgrounds and areas of expertise. All team members were working in research with expertise related to: TBI or MHSU and community integration (EN, BK, MJE, AC, VC); stigma and inequity across diverse care settings with a focus on older adults and their caregivers (PK, AG); population health and health outcomes, focusing on TBI and MHSU (VC); and TBI with diverse foci on women, sex, and gender, and underserved populations (AC). Team members were from various disciplines, including epidemiology, gerontology, and occupational therapy. Four members of the research team, who were registered occupational therapists, had previous clinical experience working in TBI and/or MHSU (MJE, BK, AC, EN).

### 2.5. Rigor

Rigor and trustworthiness in this study were established through: (1) data triangulation that involved understanding critical characteristics of housing and housing support from two perspectives (i.e., service users and service providers); (2) analytic triangulation through multiple coders and meetings with team members with diverse expertise and backgrounds that added breadth and depth to our understanding of housing and housing supports for this group; (3) prolonged engagement in data collection and analysis; and (4) maintenance of an audit trail in all stages of analysis (i.e., capturing notes regarding decisions around categories and themes, including questions generated from the analysis, tensions in perspectives, and links with existing literature) [36,37]. 

## 3. Findings

### 3.1. Participants

A total of 16 service users and 15 service providers participated in the study. Service users had an average age of 50.6 ± 6.2 years, with the majority of participants identifying as men (81.3%) and of White ethnicity (81.3%), not employed (81.8%), obtaining income from Disability Pension Plan or other social assistance (81.3%), and housed at the time of the interview (87.5%). Service provider participants had an average age of 42.8 ± 11.0 years, with many identifying as women (73.3%). There were a mix of frontline staff (60%) and managers of services (40%). The majority worked for a mental health agency and/or had more experience working with individuals with MHSU (80%) compared to those working in brain injury services or both (20%). Characteristics of service users and service providers are reported in detail in Table 1.

### 3.2. Themes

Service users experienced several barriers to finding and maintaining housing. To meet individuals’ needs, housing supports were provided holistically, in that supports were helping individuals secure and maintain housing, and also enabling engagement within their community. Two themes describe core processes involved in finding and maintaining housing for service users and the key characteristics of housing and housing supports that facilitated these processes: (1) overcoming structural barriers to housing; and (2) enabling engagement in meaningful activity and social connection. In discussing each theme, we first report barriers and needs related to these core processes, and then describe the critical characteristics of housing and housing supports from the perspectives of both service users and service providers. Participant quotes that illustrate these processes and key characteristics are reported in Table 2.

#### 3.2.1. Overcoming Structural Barriers to Housing

Structural barriers hindered service users from finding and maintaining housing. Such barriers included (1) limited housing supply and high demand, creating affordability issues; (2) attitudes towards and understanding of TBI; and (3) fragmented service systems.

Limited housing supply in the context of high demand, and affordability issues, created significant barriers to finding and maintaining housing. Limited housing supply coupled with individuals experiencing financial constraints, often due to lack of employment or history of substance use, meant market rent was often unaffordable. The demand for housing meant landlords could require higher rent, making options even more scant for service users on limited income. As a result, service users were often on long waitlists for rent-geared-to-income or affordable housing. These structural barriers also limited choice regarding housing. Service users often took the first home they were offered, either fearing they may not have another opportunity, or wanting to be out of the streets immediately. While this worked out for some, many reported being housed in places that did not suit their needs and preferences. Those who were dissatisfied with their housing environments could not easily transfer to a more suitable residence due to challenges affording market rent and the fact that they were considered “lower priority” for rent-geared-to-income housing as they were already housed.

Attitudes towards and understanding of TBI influenced access to housing and support services. Service providers reported feeling uncertain regarding their knowledge and expertise on TBI, in part because they worked in a siloed system (e.g., in mental health not in brain injury). They referred to TBI as an “unknown,” and their perceived lack of knowledge made it challenging to determine the appropriate approach to working with clients and to assess whether services being provided were effective. Service providers reported feeling ill-equipped to serve individuals with TBI, making service delivery challenging. For example, service providers described a number of behaviors, attributed to TBI, that meant complicated service delivery for these individuals such as dropping in and out of service (i.e., permanently leaving their residence without notifying anyone), anger outbursts, and “hoarding.” Cognitive difficulties were also noted and service providers reported having to explain housing processes multiple times, remind clients of conversations they had previously had, and felt uncertain about individuals’ understanding of information given to them. Poor understanding fueled stigmatizing attitudes and, in the case of landlords managing private rentals, discriminatory practices of theirs were reported, limiting access to housing and support services. When service users had access to rent subsidies and could afford market rent, their rental applications were often rejected by landlords without reason. Both service users and service providers expressed frustration over this frequent occurrence and attributed rejections to landlords discriminating against those with disability, low income, or lack of credit history. Negative attitudes, and a lack of understanding of TBI and MHSU amongst landlords, in some cases limited how service providers could offer supports to individuals in their home, as service providers discussed examples of being unable to meet with their clients in the residence.

Service providers also noted the fragmented system as influencing their ability to provide optimal services/supports, and for service users, limiting or delaying their access to required services. This fragmentation was noted between TBI and MHSU services, between hospital and community services, and between housing and healthcare services. Service users experienced this in terms of not knowing where to go for supports. Many reported feeling confused about service providers’ roles, criteria for receiving supports, and decision making with regard to accessing housing and housing services. A few reported not knowing how they settled into housing and wanted clarity on who to go to for questions.

Service providers found it challenging to work in a fragmented system, because they lacked information or had difficulty arranging additional services, and therefore could not tailor their work to their clients’ needs. For instance, siloes between hospital and community services meant service providers in community housing programs generally did not know if their clients had experienced TBI and therefore could not adapt their services accordingly. They felt that access to information on medical and service use history may be useful in determining what interventions their clients have received, what has and has not worked, and building supports around the person using that knowledge as opposed to starting from scratch. Additionally, separation of TBI and MHSU services, and also of healthcare and housing, meant that service users often had to access services from multiple systems, organizations, and providers. Service providers working in housing reported feeling unsure of what healthcare services their client may be eligible for or benefit from, and have difficulty accessing supports for their clients. They reported having to make multiple referrals to other agencies that provided clinical supports and that often their clients faced long waitlists for services (e.g., access to a counselor, psychiatrist, or brain injury or substance use specialist).

Critical characteristics of housing and supports used to overcome these structural barriers included (1) service coordination involving navigation (guiding movement/pathways to services) and facilitation (providing guidance and practical assistance); (2) education and awareness raising; and (3) partnerships and collaboration across individuals, organizations, and sectors.

Service coordination through navigation and facilitation were key to overcoming structural barriers affecting individuals’ housing situation. Navigation involved a service provider guiding the individual through systems and linking them to resources and services, while facilitation involved service providers assisting with tasks by performing the task on behalf of the individual or providing guidance. Service users described a range of navigation and facilitation supports that were helpful, including service providers assisting them in gathering requirements for housing applications and maintenance (e.g., ID and paperwork, annual tenant eligibility reviews), connecting them to financial supports to afford rent (e.g., rent subsidies, social assistance, trustee program), searching for available and preferred housing, coordinating viewings, and connecting them to affordable resources, such as food, clothing, furniture, laundry, personal care items, and in some cases technology or printed information to assist with searching for available housing. Navigation and facilitation were critical in the context of fragmented systems as service providers often had to make multiple referrals arranging required supports (clinical, health related, and/or for enabling community integration). Concerns about their client’s wellbeing and housing situation, and difficulty accessing clinical services, in some cases meant service providers felt they had to go “above and beyond” their role description to provide follow up supports that went beyond making a referral and involved active facilitation of access to services.

Education and awareness raising were viewed as important for addressing knowledge gaps and attitudes regarding TBI or MHSU. The impact of working in siloed systems was evident in what education service providers wanted. MHSU service providers wanted more education regarding TBI symptoms and indicators of recovery to know when and how to reach out to other services, and to understand when an intervention is working. Brain injury service providers recommended education for those working in mental health, regarding the physiology of the brain and which impairments may be present depending on which area of the brain is damaged to allow them to tailor their interventions to individuals’ needs. Service providers in the brain injury sector reported wanting more training on harm reduction techniques, crisis support, and de-escalation techniques, including how to support clients at risk of suicide. Notably, service providers were self-directed in building their knowledge, for example, doing their own research/reading, engaging in discussions with their team, or seeking out webinars. Many service providers emphasized the urgent need for more formal cross-sectoral education and training (i.e., provided within the system and within organizations), with the aim of having shared fundamental knowledge across sectors, in particular across TBI and MHSU. Education and awareness raising was also part of how service providers worked with communities, property managers, and landlords. Landlords commonly expressed fears that individuals would not pay rent, cause trouble with other tenants, or damage property, and service providers used education and awareness raising to counter stereotypes and stigmatizing views, and to ensure that landlords better understood the range of services available to support service users in maintaining their housing.

Partnerships and collaboration were viewed as essential for bridging fragmented systems and responding to the limited availability of affordable housing for service users. “We can’t do it alone. […] We need to partner within the community,” was a sentiment shared by many service providers from different agencies. Some service providers reported having built partnerships with property management companies and private market landlords to address structural barriers such as stigma and lack of affordable housing. In these situations, property management companies worked with a housing service to offer regular housing stock to service users. In some cases these partnerships had further benefits of increasing supports as landlords could contact the housing service for assistance if needed (e.g., if clients were not paying rent) or could access training from the housing service on specific topics (e.g., responding in a crisis). Other examples of partnerships were between individuals or organizations working in brain injury and mental health. Service providers from the mental health sector spoke about working with brain injury professionals or organizations, and vice versa, to provide specialist expertise. For example, service providers from the brain injury sector spoke about bringing in specialists from the MHSU sector for specific input related to harm reduction or substance use services. These partnerships often involved consulting with specialists from other agencies or having them be part of multidisciplinary team meetings to discuss the needs of individual clients. While promising, the examples of partnerships provided were typically localized, driven by service providers, and were not always successful, particularly in the case of working with landlords.

#### 3.2.2. Enabling Engagement in Meaningful Activity and Social Connection

Participants’ satisfaction with, and stability of, housing were shaped by their ability to engage in meaningful activity and to form social connections. Specific needs related to: (1) finding something to do with their time; (2) living independently and comfortably in housing; and (3) forming and maintaining social connections.

Service users reported difficulty in finding something to do with their time. Having limited ways to occupy their time, service users described experiencing boredom and a desire for more “structure” in their life which came from having routines, being busy, and limiting idle time. They shared that engaging in meaningful activity brought balance to their life and made them feel more positively about their home. Others who lacked meaningful activity or social connection reported that they tried to spend time away from their home as they did not get along with others in the environment, or were seeking opportunities for social connection and activity that were not available in their home environment. Other benefits of having things to do included feeling better able to cope with life circumstances, keeping their mind active, and experiencing accomplishment. Some service users also felt that engaging in activity improved their mental health, limiting the time and opportunity to dwell on their thoughts or use substances.

Living independently and comfortably in housing was important to service users and related to their ability to manage the activities required to maintain one’s home, and the feelings of safety and privacy afforded by their home and neighborhood environment. While most reported being able to manage the activities needed to maintain their home (e.g., household chores), a few reported experiencing difficulty. For example, one service user reported not being able to cook as well as they used to, while another shared that they often felt overwhelmed and did not know how to perform household chores. Service providers generally felt more strongly that individuals needed support for aspects of everyday living, including managing finances, eating consistent and healthy meals, taking medications, and managing the property. A commonly identified challenge for service users was related to managing conflict with neighbors, landlords, or others in the housing environment. Many reported that they felt unsure of how to approach conflicts in a calm way and wanted assistance, from family or service providers, in resolving tensions with others. Beyond managing the activities to maintain one’s home, some service users did not feel comfortable and safe in their home. Environments that were perceived to be unsafe were generally characterized as being noisy, lacking in privacy, and involving more unpredictable interactions with others entering or living in the residence (e.g., neighbors, guests visiting other apartments). These environments triggered feelings of anger, physical symptoms like headaches, and past experiences of trauma, and impacted participants’ daily activities and interactions with other residents. For example, one participant spoke about waking up and being on edge when another resident knocks on their door at night, and other participants were concerned for their safety and the stability of their housing when observing others using substances.

The need for social connection was articulated by all participants. Service users and service providers noted the importance of having someone to talk to, and of social interaction, in promoting mental health and in reducing stress and loneliness. Service users expressed a desire for intimacy, to meet new people, and make friends. Some expressed wanting to form new relationships, alluding to a preference for people who are moving toward recovery, using phrases such as wanting to be with “positive people,” “a community of like-minded people,” or “responsible people,” as well as wanting to be away from an environment with substance use. Despite the recognized importance of social connection, connecting with others was a challenge for many service users. Some individuals reported struggling with starting the initial connection as they feel overwhelmed. Service providers perceived that, often, service users have a sense of community in the shelter system and on the streets, and the transition of moving into housing can disrupt that community, potentially affecting the sustainability of housing. Other challenges that service providers perceived to influence the ability to form social connections included symptoms of mental illness (e.g., lack of motivation), difficulty trusting people due to experiences of trauma and abuse, difficulty communicating, and stigma affecting the beliefs and attitudes of others surrounding brain injury and mental illness.

Critical characteristics of housing and supports to enable engagement in activity and social connections included: (1) creating opportunities for engagement in meaningful activity and social interaction; (2) training and skills development; and (3) considering the design and features of home and neighborhood environments.

A critical aspect of housing supports was creating opportunities for engagement in meaningful activity and social interaction. Service providers supported service users in identifying activities they are interested in pursuing through assessments, intake forms, or checklists. Rapport building was viewed as critical to enabling engagement in activity and social connections, as there was a need to build trust in the professional–client relationship in order to support individuals in trying new activities. Once this rapport was built, service providers supported service users by creating and/or linking them to opportunities for engaging in activity and making new social connections. For example, creating employment opportunities (e.g., hiring someone to plan social events in the apartent building), linking individuals with recreational or educational activities planned by the service organization, hosting peer-led events, or linking them to day programs or other recreational programs in the community. Other strategies for enabling engagement and social connection included setting up appointments in the community to encourage service users to leave their home, accompanying them to programs until they become comfortable going on their own; providing access to transportation; and orienting them to key services in their neighborhood (i.e., where community centers or drop-in centers are located).

Service providers also supported service users through training and skills development to help them adapt to independent living. Programs or interventions reported by service providers included skills training and education for activities of daily living and instrumental activities of daily living (e.g., hygiene, cooking, laundry, budgeting or money management, transit training, meal preparation, medication, and household maintenance). In addition to training, varying levels of support were provided to help individuals manage the tasks needed to maintain their housing. For example, individuals who struggled with meal preparation could be offered meal plans and individuals who had difficulty with taking medications could be supported by having their medications delivered to them daily.

Considering the design and features of home and neighborhood environments was also important. Specifically, participants valued environments in which they feel safe, have their own space, proximity to the community, and connection to natural environments. Participants shared that having their own space gave them the freedom to do their own thing and not feel pressured to do anything they did not feel comfortable doing. Proximity to facilities that promoted activity engagement was important. Participants appreciated having areas at, or close to, the residence that were built for activities, such as a patio for cooking, a basketball court, or a billiards table. Many participants reported having communal spaces in their building but no one using them, because they were too small. This contributed to a negative atmosphere and limited opportunities to interact with other people they lived with. Service providers spoke about the need for communal spaces, such as communal dining, to allow residents to interact and get to know one another. Some service users also reported wanting to be close to existing social networks such as family and friends as well as community programs where they have the potential to meet people. Other community amenities identified as important to have close by were grocery stores, transit, and gyms. Many participants also emphasized the importance of being located near natural environments, such as parks and lakes, as these environments reduced their stress and helped them feel calm and relaxed.

## 4. Discussion

This is the first qualitative study to date that identified critical characteristics of housing and housing supports from the perspective of service users with concurrent TBI and MHSU and service providers. This study identified two core processes involved in finding and maintaining housing: (1) overcoming structural barriers; and (2) enabling engagement in meaningful activity and social connection. The critical characteristics of housing supports that enabled these processes include (1) service coordination, education and awareness raising, and partnerships and collaborations; and (2) creating opportunities, training and skills development, and design of home and neighborhood environments. This study is the first to bring together TBI and MHSU, complex co-occurring conditions, that have historically been studied separately, that has led to lack of knowledge and certainty regarding the suitability of services and unmet care needs. This study is also the first to include the views of service providers who provide housing supports for individuals with experiences of both TBI and MHSU, thereby illuminating not only service users’ valued supports but also how these supports are provided in a complex service environment in an urban Canadian location with publicly funded healthcare.

This study examined the experiences of receiving or providing housing services to individuals with concurrent TBI and MHSU and showed that systems and service providers were not well equipped to support those with concurrent conditions. Siloes between TBI and MHSU service systems contributed to difficulties accessing services, confusion for those living with TBI and MHSU about who does what in terms of their supports, and to service providers feeling that they lacked the necessary knowledge and skills to meet individuals’ needs. While barriers to housing, such as fragmented services, overlap with what has been reported in the housing literature for the broader population experiencing homelessness [8,21,38], unique findings from this study are (1) specific attitudes around TBI that affect service delivery, and (2) the nuances in how critical characteristics of housing supports are provided for individuals with concurrent TBI and MHSU.

The findings of this study support and unpack concerns raised by other researchers regarding the limited knowledge on TBI among those working in housing and the practices of moving individuals with co-occurring conditions to systems and services that are perceived to better serve them [19,20]. Service providers described their clients with TBI and MHSU as the “most complex” and also the challenges of working within the current system to try and meet their needs, causing them to either feel unable to provide services, or having to go “above and beyond” their role description. This raises critical questions about how individuals with TBI and MHSU access required services, given their experiences are often shaped by the service provider, their attitudes, knowledge, and approach to working within a complex system. While service providers noted important practices that served to address knowledge gaps from the separation of TBI and MHSU services (e.g., partnership development and education), efforts were driven by providers and therefore occurred either individually (a provider doing their own research about TBI) or at a local service level. There continues to be an urgent call for formalized education across sectors, such as those in TBI and MHSU service systems, to better understand the needs of those living with concurrent disorders and available services with opportunities for collaboration [19]. In addition to education for professionals, there is a need to consider system-level changes that may alleviate some of the pressure on service providers and better support them to provide holistic services despite systems that are more divided. For example, literature findings signal the need for integrated care which brings together design and delivery aspects of fragmented care systems to optimize care [18,39]. Emerging evidence suggests that integrated care models where one management team provides acute, rehabilitation, and community services have been found to improve functional outcomes for individuals with brain injury [40,41].

Another novel finding is the nuance in how critical characteristics of housing supports are provided for individuals with concurrent TBI and MHSU. A range of critical characteristics of housing supports were identified, including service coordination, education and awareness raising, and building partnerships, which shows the varied tasks and skill sets needed for those working in housing and serving this complex group. In fact, a tension emerged related to the Housing First approach, which calls for person-centered and holistic services and de-linking of housing and health supports. While this ensures continuity in health services if an individual loses their housing (or vice versa) [12], this also placed added importance on the service provider and their role in coordinating required services, and having the knowledge and skills to work across a range of systems and services that were established and organized according to health diagnoses (e.g., TBI and MHSU), or hospital and community settings. Often, housing supports are provided by case managers, and case management is considered critical to ending homelessness [42]. Our findings on how these critical housing supports are delivered to this group highlight the multi-faceted and complex role of case managers and the need to consider how to evaluate case management services and identify how and why services are provided given the context. For instance, in this study certain tasks were identified (e.g., assessment, navigation, facilitation, education) and these were used in different situations (e.g., responding to structural barriers or the need for meanignful activity) and with different stakeholders (individuals with TBI and MHSU, landlords). Certain skills were also identified such as rapport building and collaborative skills in building partnerships. More work to refine the range of tasks and skills, and how case management is provided, is needed to prepare service providers to take on these roles. Certain frameworks and toolkits for case management are being developed both in brain injury [43] and in relation to provision of Housing First through intensive case management [42]. Considering how these may be aligned and applied to addressing the needs of those with TBI and MHSU as it pertains to housing is encouraged.

The findings of this study identified critical characteristics of housing (i.e., meaningful activity, social connection, safety, and privacy) that are similar to what has been reported in the Housing First literature. Our findings reinforce calls in other literature to conceptualize and deliver housing services in a holistic way including enabling opportunities for meaningful activity, social connection, privacy, and security through provision of housing and supports [22,44,45,46,47]. Structural barriers to housing [8,19,48,49], the need for engagement in meaningful activity and social connection [22,23,44,50], and preferred characteristics of housing and neighborhood environments (e.g., design to promote safety, privacy, and proximity to amenities and nature) [45,46,51,52] were all consistent with what has been reported in existing literature, suggesting that these are core housing needs as opposed to needs linked to a particular diagnosis. Given that research has tended to study those with TBI and MHSU in isolation from one another [22,24,25,50], there may be benefits in future research that brings together knowledge from both fields and applying it to the issue of housing. For example, stigma was noted to be a barrier to finding and maintaining housing. Large-scale interventions to address stigma for MHSU already exist, which could be adapted to combat stigma toward individuals with TBI and tailored towards housing services and supports [53]. Similarly, service providers provided examples of how they are building partnerships with and educating landlords, to address stigma and improve access to private market housing for those with TBI and MHSU. Intervention models for communicating and working with individuals with TBI have been developed to support those in public facing roles (e.g., police officers, customer service agents, and healthcare professionals); these may be useful models for the housing context and working with landlords [54]. Alternatively, research in the area of MHSU that discusses models for working with landlords may be useful to support individuals with TBI and MHSU in maintaining their housing [55,56]. The findings of this study attest to the value of understanding the perspectives of those with concurrent TBI and MHSU and those involved in housing services (i.e., service providers), as it identified core housing needs that are not tied to diagnosis and opportunities to integrate knowledge from TBI and MHSU fields. Future research that brings together landlords, service providers, and individuals with TBI and MHSU may be useful.

Finally, housing supports, specifically supports for meaningful activity and social connection, tended to target the individual and less so the environments that shape opportunities for engagement and connection. This places the responsibility on the individual to adapt or develop skills to meet their needs. Critical characteristics of housing supports included creating opportunities for engagement in activity and social interaction and training and skills development (e.g., in medication management, financial management). Equally important, however, would be a focus on intentionally designing environments to consider service users’ preferred characteristics of housing (e.g., safety, privacy, having their own space, proximity to the community, and connection to natural environments). There are a few studies conducted in the United States that summarize insights regarding design of housing and community spaces for individuals experiencing homelessness [57,58,59]. These studies also highlight the potential of intentionally designed, trauma-informed architecture, given the influence of built environments on subjective well-being, human connection, and community participation [59]. Our findings suggest that, at present, service users and providers utilize strategies to adapt to housing and neighborhood characteristics. Given the growing demand for housing and unmet housing needs of individuals with TBI and MHSU, the need to involve service providers and individuals with TBI and MHSU in the planning and design of built spaces is evident.

### Strengths, Limitations, and Future Directions

A core strength of this study is bringing together multiple perspectives and obtaining an in-depth understanding of the service context to illuminate not only the critical characteristics of housing suppports but how and why they are provided. A limitation to the study was the lack of diversity among participants. For instance, those with TBI and MHSU predominantly identified as men and White, whereas service providers mostly identified as women and White. Future research that includes a more diverse sample (i.e., gender, race/ethnicity, class) and uses an intersectional lens is needed to understand how social identities shape individuals’ experiences of housing and housing supports. Additionally, interviews occurred prior to the COVID-19 pandemic and did not capture the complexities of providing services during the pandemic as well as novel housing supports that may have developed as a result of the pandemic. Future directions include implementing formalized education and housing research on TBI and exploring system-level solutions on how to best support service providers in overcoming structural barriers. Findings point to certain interventions that may hold promise for addressing structural barriers and promoting engagement in meaningful activity and social connection. Some interventions discussed earlier that could be further researched in this context include large-scale stigma interventions, communication partner training to support those working with individuals with TBI and MHSU (e.g., landlords), models of integrated care, and intentional design and trauma-informed architecture. Alongside research, policy analyses may be beneficial in considering how policies respond to the needs of those with TBI and MHSU in finding and maintaining housing, and enable services to operate in ways that address structural barriers and enable engagement in meaningful activity and social connection.

## 5. Conclusions

Services providers are challenged in supporting individuals with concurrent TBI and MHSU in finding and maintaining housing, in part due to structural barriers and siloed systems. Structural barriers hindered service providers from delivering care, and individuals with TBI and MHSU needed more support to engage in meaningful activity and form social connections to stay housed. Service coordination, education and awareness raising, partnerships and collaboration, creating opportunities for engagement in meaningful activity and social connection, training and skills development, and design of home and neighborhood environments are critical housing supports that work well. There is an urgent need for formalized education across sectors, supports to prepare service providers to take on case management roles, and consideration of potential interventions that draw from TBI and MHSU expertise to overcome structural barriers, promote engagement in meaningful activity and social connection, and ultimately enable greater housing stability for individuals with concurrent TBI and MHSU.

## Figures and Tables

**Table 1:** Participant characteristics.

Service Users (*n* = 16)		Service Providers (*n* = 15)	
Demographic Characteristics	Total N (%)	Demographic characteristics	Total N (%)
Age, mean (SD)	50.6 (6.2)	Age, mean (SD)	42.8 (11)
Gender		Gender	
Man	13 (81.3)	Man	4 (26.7)
Woman	2 (12.5)	Woman	11(73.3)
Other	1 (6.3)	Education	
Education		Post-secondary degree	7 (46.7)
Grade eight	2 (12.5)	Some post-graduate work	2 (13.3)
Some high school	5 (31.3)	Post-graduate degree	6 (40)
High school graduate	2 (12.5)	Job Category	
Some college/post-secondary	3 (18.8)	Frontline (e.g., housing worker, crisis support worker, case manager)	9 (60)
Post-secondary degree	3 (18.8)	Management (e.g., program coordinator, housing manager, clinical manager)	6 (40)
Some post-graduate work	1 (6.3)	Experience in Population	
Marital Status		0–2 years	1 (6.7)
Single, never married	8 (50)	3–4 years	3 (20)
Married or domestic partnership	1 (6.3)	5–9 years	3 (20)
Divorced	5 (31.3)	10+ years	8 (53.3)
Separated	2 (12.5)		
Ethnicity			
White	13 (81.3)		
Black	2 (12.5)		
Asian	1 (6.3)		
Living Situation			
Living alone	8 (50)		
Living with others	5 (31.3)		
Shelter/Other	2 (12.5)		
Mental Health and/or Substance Use Diagnosis ^a^			
Schizophrenia and other psychosis	2 (12.5)		
Depression	3 (18.8)		
Anxiety	2 (12.5)		
PTSD	3 (18.8)		
History of substance use	3 (18.8)		
No diagnosis reported but receiving mental health supports	5 (31.3)		
Traumatic Brain Injury			
Diagnosed	8 (50)		
Self-reported	8 (50)		
Mechanism/Context of Brain Injury ^b^			
Assault	9 (56.3)		
Fall	5 (31.3)		
Vehicular accident	3 (18.8)		
Struck by/against	1 (6.3)		
Sports	1 (6.3)		

^a^ The total number of participants reporting mental health and/or substance use challenges (MHSU) may not total to 16, as an individual can have more than one MHSU status. ^b^ The total number of participants that reported mechanism/context of brain injury will not add up to 16, as an individual can have more than one mechanism of injury.

**Table 2:** Themes, sub-themes, and sample quotes.

Theme	Sub-Theme	Quotes
Overcoming structural barriers to housing	Limited housing supply in the context of high demand and affordability issues	[…] But they recognize, you know, the mental health challenges…well, my shortcomings and the challenges the house presents to me, and how that affects my housing. A few of them have said you’ve got to move. If you can’t sleep, you can’t stay there. So I’m in the odd situation that I finally have long term stable housing but it’s almost like it’s not healthy for me so I’ve got to keep looking […] and then the challenge there, and this might be a systemic challenge, so okay, you’re in long term stable housing, subsidized. Why should we take you when there’s a homeless person applying for the same place?—Service user
	Attitudes towards and understanding of TBI	But certain landlords won’t even open the door for you. And you can explain… I’ve been in a situation where I was like, “Oh, hi. I’m so and so. I’m a case manager. I’m here to see this client. Can you please let me know… They’re expecting me.” It’s like, “No, she has to open the door herself.” … When I got access into the building, what I walked into was the client laying down in a pool of blood because she fell and cracked her head open. And had to go to the hospital and get 12 stiches across her forehead because she was drinking and she fell. …So it goes both ways. It’s like we’ve had lives saved because of great landlords who were very supportive, and could have potentially had lives lost because of the difficult landlords—Service provider
		But it’s very hard to convince those landlords to take people that are not working because they can easily get $1000 for the room from a person who’s working […] There’s many reasons […] but the stories I hear from the landlords [are], “No, the last guy I had in here was in here drinking and had all his buddies here, and keeping me and my wife up all night. And I don’t want to have another person in my basement like that.” […] Or “No, I don’t want someone who’s home all day and all night because they’re going to use all my hydro.” You know, they have all these reasons. But it’s hard, you know. […] Like with the rent [supplement], we can negotiate. We can say, “Yes, but they can afford now to pay for the hydro.” So there’s a little buffer when you have a rent [supplement] When you don’t have a rent [supplement], it’s next to impossible. They would much rather get a student who’s paid direct from one of the student placement agencies. So there’s such competition out there right now.—Service provider
		So after a while, he just disappeared from the house. He left his key there and he told his housemates he was leaving. So I think this person will end up homeless again just because he didn’t really have any… Well, I think he had supports but he would not accept, right. He just decided… And again, I don’t know if he decided or because he was confused. I don’t know if he knows what he was doing. But I believe he will wind up again homeless just because of his confusion. So in this situation, it’s hard to provide support. Like even though support is there, the person is not accepting. There is not much we can do.—Service provider
		What happens is I found 1) they come in and out of service because of the way the service is provided. It’s not the service itself, it’s the way the service is provided. Because people with a brain injury require different methods on how you engage them, on how you work with them, and how you continue to work with them and how you follow-up with them. […] So their social skills are very bad usually because almost all brain injuries are frontal lobe. So you end up with people with poor social skills. They’re rude, they’re loud, they’re aggressive, they fly off the handle really easily. They don’t understand. And people don’t take the time in order to work through those pieces to help people who have a brain injury. Because they don’t know they have a brain injury. They don’t understand. They can’t identify it. And so giving service providers education on how to help somebody with a brain injury, identify somebody with a brain injury, you’re miles ahead of what’s happening right now.—Service provider
	Fragmented system	I didn’t know anything. I didn’t know all the rules. I’d hear little tidbits from say [social worker] or whoever. But I’m like I just… I don’t know, I just felt like I was on my own. […] So I don’t know, like knowing who to talk to and knowing what to say and what to ask for, etc. Things like that.—Service user
Critical characteristics of housing supports	Service coordination through navigation and facilitation	Well, I’ve had situations where I can sense that somebody is not doing well. So I try to help them finding let’s say a case worker or a case manager or… Yeah, I’ve gone above and beyond my job description to try to help someone maintain their housing […] in this case, I had to go through the Access Point application process, to fill out for her. So that’s something I can do. It’s in my job description. But beyond that, I went with her to doctor’s appointments to make sure she was attending. I went with her to the case worker’s meetings to make sure she would go. So those things I went beyond. So I had to ask my manager for permission. Because I was aware that if I didn’t go with her, she would probably miss the appointment and that would cause her…you know, there would be consequences. So that was something I did beyond my job description.—Service provider
	Education and awareness raising	I thought like I’m really under-resourced in terms of expertise. So we don’t know… What is it that I don’t know–it’s a hard question to answer. […] I think it would just be have more of an awareness. Like I know mental health, there’s illnesses, for example, and I know kind of the symptoms of those illnesses. So when a person presents this way, chances are it could be this or it could be that. You have a frame of reference. With [TBI], I don’t have that. I really don’t know what’s a sign, what do I look for to know that things are getting worse, things are getting better. Like I don’t know what that baseline is and what those points of reference are.—Service provider
	Partnerships and collaboration	Well, I think part of the fragmentation is caused by the system. I think how we’re funded is an issue. I think how agencies are structured is an issue […] But in order to move forward, we need to create real partnerships and we need to work together to mitigate like fragmented treatment outcomes […] So as an example, with one of the clients who has severe cognitive impairments, he was receiving community-based addiction support. Which is great. But he was getting nothing from it because of the cognitive impairments. So it was only when we would both literally be there together, working side by side, were we actually able to obtain any results.—Service provider
Enabling engagement in meaningful activity and social connection	Finding something to do with their time	I wish I had help more with like having a schedule like during the day. Like things to keep myself occupied. […] I think it would help me a lot because I’d have things I’ve got to do every day. If you’ve got a schedule, okay, I’ve got to do this, I’ve got to do this, I’ve got to do this, I don’t have time to sit at home and look at the wall and do drugs or do…you know, drink and stuff. If I’ve got my time occupied, I don’t have time to do that kind of stuff, right. Because if you’ve got a schedule, it keeps you in line, right. It keeps you, okay, I’ve got this I’ve got to do, I’ve got that that I’ve go to do. You’re not going to have time to be doing stupidity. That’s what I need, is a schedule.—Service user
	Living independently and comfortably in housing	But they need a safe place. They need a place where they don’t have to look over their shoulders, where they don’t have to hear their neighbour screaming at their son or something like that. You know, that’s really the biggest thing. Loud noise is not a helpful thing for anybody with a brain injury […] So that’s the whole thing about it, is they’ve got to have a safe place.—Service user
	Need for social connection	So I don’t know, the home? Just a nice, safe, quiet, comfortable location but also amongst people of like mind. Like a community.—Service user
Critical characteristics of housing supports	Creating opportunities for engagement in activity and social interaction	And another thing I think the team does well is supports people going to programs. So like they’ll walk them to the community centre. We often need like maybe 10 times with that warm handover and then finally the client will start going on their own. But taking the time to sort of…to go with them and integrate into different community programs, I think is […] something that’s really important–in supporting people in connecting with the different like community social, recreational activities—Service provider
	Training and skills development	So that’s life skills, right. So that’s understanding how to be clean. Like you can’t pluck somebody from the street and have an expectation that they’re going to know how to cook, and they’re going to know how to clean, and they’re going to know how to budget. So we offer them life skills in the ADLs. So what we call activities of daily living […] So understanding how to budget, you know, and how to budget food. You know, you make leftovers, and this is how you can cook […] So we do those things.—Service provider
	Design and features of home and neighborhood environments	So our building […] feels like a home. It doesn’t feel like… You know, it doesn’t feel temporary. It doesn’t feel like a hospital, you know. And I think that, you know, having access to privacy, having access to security, those are really important things that clients highlight when they come. You know, like having privacy is…like they really love that. And knowing that they’re in a safe place. Those are two really, really important things.—Service provider

## Data Availability

The data presented in this study are available on request from the corresponding author. The data are not publicly available due to privacy restrictions.
